# Nitrogen metabolism and mammary gland amino acid utilization in lactating dairy cows with different residual feed intake

**DOI:** 10.5713/ab.20.0821

**Published:** 2021-02-15

**Authors:** Yunyi Xie, Chao Miao, Yi Lu, Huizeng Sun, Jianxin Liu

**Affiliations:** 1 Institute of Dairy Science, MOE Key Laboratory of Molecular Animal Nutrition, College of Animal Sciences, Zhejiang University, Hangzhou 310058, China

**Keywords:** Amino Acid Metabolism, Mammary Gland, Nitrogen Metabolism, Residual Feed Intake

## Abstract

**Objective:**

This study was conducted to enhance our understanding of nitrogen (N) metabolism and mammary amino acid (AA) utilization in lactating cows with divergent phenotypes of residual feed intake (RFI).

**Methods:**

Fifty-three multiparous mid-lactation Holstein dairy cows were selected for RFI measurements over a 50-d experimental period. The 26 cows with the most extreme RFI values were classified into the high RFI (n = 13) and low RFI (n = 13) groups, respectively, for analysis of N metabolism and AA utilization.

**Results:**

Compared with the high RFI cows, the low RFI animals had lower dry matter intake (p<0.01) with no difference observed in milk yield between the two groups (p> 0.10). However, higher ratios of milk yield to dry matter intake (p<0.01) were found in the low RFI cows than in the high RFI cows. The low RFI cows had significant greater ratios of milk protein to metabolizable protein (p = 0.02) and milk protein to crude protein intake than the high RFI cows (p = 0.01). The arterial concentration and mammary uptake of essential AA (p<0.10), branched-chain AA (p<0.10), and total AA (p<0.10) tended to be lower in the low RFI cows. Additionally, the low RFI cows tended to have a lower ratio of AA uptake to milk output for essential AA (p = 0.08), branched-chain AA (p = 0.07) and total AA (p = 0.09) than the high RFI cows.

**Conclusion:**

In summary, both utilization of metabolizable protein for milk protein and mammary AA utilization are more efficient in cows with lower RFI than in the high RFI cows. Our results provide new insight into the protein metabolic processes (related to N and AA) involved in feed efficiency.

## INTRODUCTION

Improving protein efficiency is an established goal in the dairy industry, as it is expected to increase profits and environmental benefits. Residual feed intake (RFI) has been a popular indicator of feed efficiency in recent studies [1,2] and is calculated as the difference between the actual and predicted dry matter intake (DMI) [3]. Accumulating evidence indicates that cows with lower RFI have higher efficiency of utilization of protein or nitrogen (N) [4,5]. However, the underlying mechanism is not clear.

Metabolism of N or amino acid (AA) is an important biological process. It is strongly associated with N efficiency and may directly affect RFI. Animals with low RFI (LRFI) consume less dry matter (DM) than high RFI (HRFI) animals [6]. Studies show that microbial protein (MCP) synthesis increases with increasing levels of feed intake [7]. However, increased feed consumption may decrease nutrient digestion in the rumen. Both MCP and metabolizable protein (MP) are vital to the lactation of dairy cattle [8]. In our previous study, we found no significant difference in rumen fermentation parameters or MCP production between LRFI and HRFI cows [9], while cows with LRFI values had higher ratio of milk protein to crude protein (CP) intake. Thus, the steps of MCP utilization for milk production, including the efficiencies of conversion of MCP to MP and of MP to milk, may potentially contribute to the greater N efficiency in LRFI cows. It is hypothesized that variation exists between LRFI and HRFI cows in MCP and MP utilization.

The N efficiency of dairy cows may be improved by increased utilization of AA in mammary gland [10]. However, information regarding AA utilization in mammary gland by lactating cows of differing RFI is lacking. Measurements of AA concentrations and mammary plasma flow (MPF) may provide an indication of AA utilization in mammary gland. Therefore, the objective of the current study was to investigate the relationships between RFI and MCP synthesis or mammary AA utilization in mid-lactation dairy cows. Understanding the utilization of N and AA in lactating cows with divergent RFI may provide a new insight into metabolic differences among cows differing in RFI and is essential to effectively utilize RFI in production systems.

## MATERIALS AND METHODS

### Animals and management

The animal care and experimental procedures were approved by the Animal Use and Care Committee of Zhejiang University (Hangzhou, China, No. 12410). Fifty-three multiparous mid-lactation Holstein cows, with body weight (BW) of 634 (±85 kg, standard deviation) and days in milk of 153 (±20, standard deviation) were selected for the experiment. The experiment lasted for 57 d, including a 7-d period for adaptation. During this period, the DMI of each cow was determined daily by an automatic feed system (Zhenghong Co., Shanghai, China). All cows were housed in a free-stall barn with access to a total mixed ration, and were fed three times a day (06:30, 14:30, and 21:30). Animals were weighed immediately after the morning milking every week.

### Sampling and measurements

#### Feed

Samples of diet were collected once a week and frozen at −20°C for later analysis. The samples were dried at 65°C for 48 h, ground through a 1-mm mesh, and analyzed for DM (105°C for 5 h), CP (method No. 988.05), ash (method No. 942.05), neutral detergent fiber (method No. 2002.04), and acid detergent fiber (method No. 973.18) [11]. The chemical composition of the diets is listed in Table 1.

#### Milk

The milk yield of each cow was recorded at each milking (06: 00, 14:00, and 21:00). Each week, milk samples were obtained on 2 consecutive days at each milking and then pooled. One subsample was stored at 4°C for analysis of fat, lactose, protein, somatic cells, and urea N with infrared spectroscopy (Foss-4000; Foss Electric A/S, Hillerød, Denmark). Another subsample was frozen at −20°C for analysis of AA.

#### Urine

Spot urine samples were collected from each cow 3 times daily (05:00, 13:00, and 20:00) for 2 consecutive days (d 49 to 50). Urine samples were acidified with 0.036 mol/L H_2_SO_4_ (1:4, v/v) and then frozen (−20°C) for later analysis of allantoin, uric acid, creatinine [12], and urea N [13]. Urinary excretion of purine derivatives (PD) was used to estimate MCP yield in the rumen [12].

#### Analysis of plasma and milk amino acid

Blood samples from coccygeal artery and the subcutaneous abdominal vein were collected from each cow for two consecutive days at approximately 07:00, 15:00, and 22:00. Blood was immediately put on ice until centrifugation (3,000×g) at 4°C for 15 min), and the plasma was stored at −20°C for later analysis. The pooled plasma was analyzed for AA by previously described methods [14]. Briefly, an aliquot of 1 mL of plasma was deproteinized with 10% sulfosalicylic acid (1:1, plasma to 10% sulfosalicylic acid). Samples were then centrifuged at 10,000×g at 4°C for 15 min. The supernatant was filtered through 0.45 μm and 0.22 μm nylon syringe filter units (Fisher Scientific, Pittsburgh, PA, USA) and placed in microcentrifuge tubes (Fisher Scientific, USA). Before analysis, milk was hydrolyzed by adding 6 *N* HCl and incubating at 110°C for 24 h [11]. The AA concentrations of plasma and milk were analyzed using an automatic AA analyzer (Hitachi High-Technologies Corporation, Tokyo, Japan).

### Calculations

#### Residual feed intake

The RFI value of each animal was calculated as described previously [9]. Briefly, the RFI was estimated as the difference between expected feed intake and actual feed intake. Expected feed intake was computed using a multiple linear regression model, regressing DMI on measures of energy-corrected milk (ECM) yield, metabolic BW (BW^0.75^), and average daily gain (ADG) over the measurement period:

$${\text{Y}}_{1}=\alpha_{0}+\alpha_{1}\;{\text{ECM}}_{1}+\alpha_{2}\;{{\text{BW}}^{0.75}}_{1}+\alpha_{3}\text{ADG}+{\text{e}}_{\text{i}},$$

where, Y_i_ is the average DMI of the *i*th animal, α_0_ is the intercept, α_1_, α_2_, and α_3_ are the partial regression coefficients for ECM, BW^0.75^, and ADG, respectively, and e_i_ is the random error associated with the *i*th animal. The ADG was calculated as the slope from the regression of BW in the experimental period. All animals were ranked by RFI. With a power of 99% for RFI, the 13 lowest RFI and 13 highest RFI cows were selected to form two RFI groups: LRFI (high efficiency, n = 13) and HRFI (low efficiency, n = 13).

#### Microbial protein and metabolizable protein

The MCP was estimated from PD excretion in urine [12]. Creatinine has been validated as a marker to estimate urine volume and is assumed to be excreted at a rate of 29 mg/kg of BW [15] for calculating the urine volume excretion rate. The MP was calculated as the sum of the intestinally absorbable dietary protein (IADP) and intestinally absorbable MCP (IAMCP). The IADP was calculated as follows: IADP = rumen undegraded protein (RUP) content × CP intake × IDP, where IDP is the intestinal digestibility of RUP, determined according to the three-step *in vitro* procedure [16]. The IAMCP was calculated using the equation IAMCP = MCP×0.64 [8].

#### Amino acid utilization

The MPF was estimated using the Fick principle [17]. The equation was as follows:

MPF (L/d)=(milk Phe+Tyr) (g/d)×0.965/(arteriovenous difference Phe+Tyr) (g/L)

The indices reflecting mammary AA utilization were calculated as follows:

$$\begin{array}{l} \text{Mammary uptake }(\text{g}/\text{d})=\text{MPF }(\text{L}/\text{d})\times \text{arteriovenous difference }(\text{AVD},\text{g}/\text{L})\\ \text{U:O ratio}=\text{mammary AA uptake }(\text{g}/\text{d})/\text{milk AA output }(\text{g}/\text{d}) \end{array}$$

### Statistical analysis

The variation of the data in this study was analyzed using the general linear model procedure of SAS according to the model Y_i_ = μ+β_i_+e, where Y_i_ is the dependent variable, μ is the overall mean, β_i_ is the fixed effect of RFI group, and e is the residual error. Significance was considered at p≤0.05, and a tendency was defined as 0.05<p≤0.10.

## RESULTS

### Feed intake and lactation performance

The DMI and productivity of the cows selected for high or LRFI are presented in Table 2. The cows with lower RFI values had lower DMI than the HRFI ones (p<0.01) but greater ratios of milk to DMI (p<0.01) and ECM to DMI (p<0.01). The cows with lower RFI values had lower milk urea N than the HRFI cows (p = 0.05). The percentages of milk fat (p = 0.04) and milk protein (p = 0.05) were lower in cows with lower RFI, but the yield of milk, milk fat and milk protein were similar between the two groups (p>0.10).

### Production of microbial protein and metabolizable protein

The urinary PD data, estimated MP yield and MP utilization efficiency are shown in Table 3. No difference was found between the two groups in urinary PD (p = 0.21), MCP yield (p = 0.21) or MP supply (p = 0.23). The LRFI cows had lower IADP (p = 0.04) but higher proportion of MP to milk protein (p = 0.02) and greater ratio of milk protein to CP intake than the HRFI group (p = 0.01, Table 3). However, the partitioning of RDP to MCP (p = 0.64) and dietary protein to MP (p = 0.73) was not different between the two groups.

### Mammary utilization of amino acid

The arterial concentrations of leucine (p = 0.07), Val (p = 0.08), essential AA (EAA, p = 0.06), branched-chain AA (BCAA, p = 0.10) and total AA (p = 0.07) tended to be lower in the LRFI cows than in HRFI cows (Table 4). The arterial supply of all AA was not affected by RFI (p>0.10). The LRFI cows tended to be lower in AVD of leucine (p = 0.07), lysine (p = 0.08), EAA (p = 0.07), BCAA (p = 0.10) and total AA (p = 0.10, Table 4), but no difference was observed in the other AA between two groups. The MPF did not differ between the two groups (p>0.10).

The cows with lower RFI values had lower mammary up take of non-essential AA (NEAA), including cysteine (p = 0.03, Table 5) and proline (p = 0.05), and tended to have lower uptake of leucine (p = 0.09), lysine (p = 0.09), threonine (p = 0.10), valine (p = 0.09), EAA (p = 0.09), BCAA (p = 0.09), group 2 AA (sum of arginine, valine, isoleucine, leucine, lysine, and threonine, p = 0.09) and total AA (p = 0.09) than the HRFI cows (Table 6). The LRFI cows had lower ratio of mammary uptake to milk protein output (U:O ratio) for cysteine (p = 0.02, Table 5) and proline (p = 0.05). The U:O ratio for leucine (p = 0.08), valine (p = 0.09), proline (p = 0.05), EAA (p = 0.08), BCAA (p = 0.07), group 2 AA (p = 0.07) and total AA (p = 0.09, Table 5) tended to be lower in the cow with lower RFI values than the cows in HRFI group.

## DISCUSSION

Investigation of the conversion efficiencies of dietary CP into MP and of MP to milk protein among cows with divergent feed efficiency is instrumental for improving animal N efficiency. The lower IADP observed in the LRFI group in our study can be attributed to the lower consumption of feed in this group. The MCP, RUP and endogenous CP contribute to the passage of MP to the tissue, and MCP accounts for the majority of the MP flow [8]. However, we found no difference in MCP yield between the two groups, which may have contributed to the similar MP supply between the groups. A higher ratio of milk protein to MP was found in the LRFI animals, which consumed less DM. Our results are consistent with Rius et al [18], who reported greater N utilization and efficiency of MP in cows with lower N intake. The higher ratio of milk protein to MP in the LRFI cows indicated more efficient utilization of AA in mammary gland in these cows.

Absorbed AA are provided by MCP, and RUP is vital as a precursor for protein synthesis. The AA utilization in mammary gland is high but varies among individual animals [19], indicating the potential for improvements of AA utilization efficiency in mammary gland. However, few studies have reported the relationship between efficiency of mammary AA utilization and RFI. Many studies have been conducted to improve AA utilization efficiency through nutritional manipulation, such as infusing AA or hormones [20,21] and altering dietary levels of energy or protein [22]. In comparison with nutritional manipulation, genetic selection may provide cumulative, longer-term enhancements of traits [23]. Therefore, the relationship between RFI and AA utilization efficiency in mammary gland warrants investigation.

The AA availability, AA uptake and protein synthesis are factors regulating AA utilization in mammary gland [19]. Doepel and Lapierre [20] reported a negative relationship between MPF and EAA supply, suggesting a compensation mechanism of EAA and blood flow in the mammary gland. In the current study, the content of arterial plasma free AA tended to be lower in the LRFI cows, corresponding to the lower DMI in this group. Our results are consistent with Martineau et al [24], who reported that a low MP supply decreased the concentration of AA. However, the supply of arterial plasma AA to mammary gland was similar between the HRFI and LRFI animals, a finding possibly attributable to the similar MPF between the groups.

In mammary epithelial cells, BCAA is a potential source of energy for highly active metabolic processes and nitrogenous precursors for the NEAA synthesis [25]. Studies have shown that BCAA plays a vital role in cell signaling. Specifically, leucine and Isoleucine can stimulate protein synthesis with the phosphorylation of mammalian target of rapamycin [26]. Thus, a U:O ratio of BCAA higher than 1.00 indicates its potential use for NEAA synthesis and regulatory signals. Although we observed no difference in milk yield between the RFI groups, BCAA uptake was higher in the HRFI cows. Several studies have demonstrated a decreased efficiency of mammary utilization of AA and increased catabolism of AA when the supply of MP or AA is increased [27,28]. Thus, the higher uptake of BCAA in the HRFI cows and the higher DMI in this group could be attributable to higher BCAA catabolism in this group. However, the molecular mechanisms underlying the difference in AA utilization in mammary gland between cows of differing RFI could not be determined from our study. Recent studies have reported that transcriptomics is a powerful tool to investigate gene expression related to feed efficiency [29,30]. Thus, further research using transcriptomics is needed to investigate the regulatory genes and molecular mechanisms underlying the difference in mammary AA utilization among cows of divergent RFI.

In conclusion, the results of the current study revealed variation in N utilization and AA utilization efficiency in mammary gland in lactating dairy cows with divergent RFI values. The cows with lower RFI values had lower DMI than, but similar milk yield to the higher RFI cows. Despite similar MCP and MP production between the groups, the ratio of milk protein to MP was higher in the lower RFI cows, which is consistent with the higher efficiency of AA utilization by mammary gland observed in this group. Further research is needed to reveal the underlying mechanisms.

## Figures and Tables

**Table 1:** Ingredients and nutrient composition of total mixed ration fed to the cows throughout the experiment

Items	
Ingredient (% as DM basis)	
Alfalfa hay	12.7
Oat hay	7.50
Corn silage	21.0
Brewer’s grains	2.75
Beet pulp	6.5
Cottonseed meal, whole	7.05
Corn grain, ground	21.5
Double-low rapeseed meal	1.34
Soybean meal	11.6
Expanded soybean	2.83
Fat meal	0.87
CaHPO_4_	0.31
NaCl	0.48
Limestone	0.86
NaHCO_3_	0.86
MgO	0.29
Premix^1)^	1.53
Nutrient (% of DM)	
DM	51.4
Organic matter	95.5
Neutral detergent fiber	31.8
Acid detergent fiber	19.0
CP	17.1
RDP (% of CP)	67.9
RUP (% of CP)	32.1
NE_L_ (Mcal/kg of DM)	1.79

DM, dry matter; CP, crude protein; RDP, rumen degradable protein; RUP, rumen undegraded protein; NEL, net energy for lactation.

1) Premix, formulated to provide (per kg of DM): vitamin A ≥600 KIU, vitamin D_3_ ≥150 KIU, vitamin E ≥2,000 IU, nicotinic acid ≥500 mg, Cu ≥1,500 mg, Fe ≥1,500 mg, Mn ≥1,500 mg, Zn ≥7,000 mg, I ≥90 mg, Se ≥50 mg, Co ≥20 mg.

**Table 2:** Milk production and feed efficiency in lactating cows selected for phenotypic divergence in residual feed intake

Items	Low RFI	High RFI	SEM	p-value
DMI (kg/d)	24.4	27.1	0.54	<0.01
Milk yield (kg/d)				
Milk	34.9	34.0	1.29	0.66
ECM^1)^	37.3	37.6	1.22	0.87
Milk composition				
Fat (kg/d)	1.41	1.44	0.04	0.59
Fat (%)	4.02	4.27	0.08	0.04
Protein (kg/d)	1.12	1.14	0.04	0.80
Protein (%)	3.23	3.34	0.04	0.05
Lactose (kg/d)	1.75	1.68	0.07	0.48
Lactose (%)	5.01	4.94	0.03	0.11
MUN (mg/dL)	13.1	14.3	0.39	0.05
Feed efficiency				
Milk/DMI (kg/kg)	1.42	1.25	0.03	<0.01
ECM/DMI (kg/kg)	1.52	1.39	0.02	<0.01
RFI (kg/d)	−1.37	1.37	0.14	<0.01

RFI, residual feed intake; SEM, standard error of the mean; DMI, dry matter intake; ECM, energy-corrected milk yield; MUN, milk urea nitrogen.

1) ECM = (0.3246×kg of milk)+(13.86×kg of milk fat)+(7.04×kg of milk protein).

**Table 3:** The urinary purine derivatives and estimated metabolizable protein supply to the dairy cows selected for phenotypic divergence in residual feed intake

Items	Low RFI	High RFI	SEM	p-value
Urine volume^1)^ (L/d)	35.3	39.5	2.64	0.24
Urinary PD (mmol/d)				
Allantoin	516	553	24.4	0.29
Uric acid	54.5	66.6	6.49	0.20
Endogenous PD	54.1	52.7	1.15	0.38
Sum	514	567	28.9	0.21
MCP^2)^ (g/d)	2,746	3,030	154	0.21
IADP^3)^ (g/d)	748	825	25.2	0.04
MP^4)^ (g/d)	2,522	2,736	122	0.23
MCP/RDP (%)	88.6	85.6	4.52	0.64
MP/CP intake (%)	63.5	62.5	2.22	0.73
Milk protein/MP (%)	45.4	40.4	1.38	0.02
Milk protein/CP intake (%)	28.3	25.6	0.69	0.01

RFI, residual feed intake; SEM, standard error of the mean; PD, purine derivatives; MCP, microbial protein; IADP, intestinally absorbable dietary protein; MP, metabolizable protein; RDP, rumen degradable protein; CP, crude protein; RUP, rumen undegraded protein; IAMCP, intestinally absorbable MCP.

1) Urine volume (L/d) = BW (kg)×29 (mg/d)/creatinine (mg/L)[15].

2) MCP was indirectly estimated by the following equation [12]: MCP = (allantoin + uric acid – endogenous PD)×70×6.25/(0.85×0.116×0.83×1,000).

3) IADP = RUP×CP intake×IDP, where IDP is the measured intestinal digestibility of RUP.

4) MP = IAMCP+IADP.

**Table 4:** Arterial plasma concentration and arteriovenous difference of amino acid, and mammary plasma flow in lactating cows selected for phenotypic divergence in residual feed intake

Items	Arterial plasma		SEM	p-value	AVD		SEM	p-value
	
	Low RFI	High RFI			Low RFI	High RFI		
EAA (mg/L)	172	188	5.85	0.06	49.9	61.9	4.41	0.07
Arginine	23.8	23.4	0.89	0.75	6.69	7.19	0.51	0.50
Histidine	9.66	10.7	0.56	0.22	2.02	2.43	0.18	0.12
Isoleucine	17.0	18.7	1.01	0.26	6.09	7.53	0.65	0.13
Leucine	22.3	26.2	1.44	0.07	8.66	11.2	0.92	0.07
Lysine	14.0	15.3	0.71	0.20	7.02	8.61	0.61	0.08
Methionine	3.44	3.53	0.16	0.68	2.06	2.41	0.16	0.13
Phenylalanine	10.5	11.3	0.65	0.40	2.78	3.55	0.50	0.29
Threonine	38.0	40.6	1.45	0.22	8.08	10.7	1.24	0.18
Valine	33.1	38.5	2.12	0.08	6.54	8.33	0.82	0.14
NEAA	103	106	3.12	0.54	15.7	18.4	1.77	0.29
Alanine	25.1	23.8	1.01	0.37	2.74	3.03	0.43	0.67
Aspartate	1.57	1.76	0.20	0.52	−0.03	−0.02	0.24	0.97
Cysteine	4.45	3.95	0.39	0.40	0.04	0.12	0.06	0.32
Glutamate	19.4	21.1	0.80	0.16	5.38	5.16	0.51	0.76
Glycine	22.7	23.8	1.58	0.66	−0.29	0.35	0.56	0.44
Proline	10.8	11.5	0.59	0.43	1.48	1.82	0.28	0.41
Serine	8.65	8.77	0.30	0.79	3.21	3.57	0.25	0.31
Tyrosine	10.6	11.5	1.02	0.55	3.19	4.40	0.56	0.14
BCAA (mg/L)	72.5	83.4	4.51	0.10	21.3	27.0	2.36	0.10
Total AA (mg/L)	275	294	7.17	0.07	65.7	80.3	5.74	0.08
MPF1) (L/d)	15,348	15,152	2,379	0.95	-	-	-	-

AVD, arteriovenous difference; AA, amino acid; RFI, residual feed intake; SEM, standard error of the mean; EAA, essential AA; NEAA, non-essential AA; BCAA, branched-chain AA; MPF, mammary plasma flow.

1) MPF (L/d) = (milk phenylalanine + tyrosine) × 0.965/[arteriovenous difference of (phenylalanine + tyrosine)] [17].

**Table 5:** Uptake of amino acids and ratio of uptake to output of amino acids in lactating cows selected for phenotypic divergence in residual feed intake

Items	Uptake^1)^ (g/d)		SEM	p-value	U:O ratio		SEM	p-value
	
	Low RFI	High RFI			Low RFI	High RFI		
EAA	665	833	64.0	0.09	1.49	1.79	0.11	0.08
Arginine	85.7	99.8	7.83	0.24	2.80	2.99	0.23	0.42
Histidine	27.2	33.5	2.85	0.17	1.12	1.22	0.11	0.26
Isoleucine	82.8	101.3	8.03	0.14	1.80	2.09	0.13	0.12
Leucine	117	150	12.4	0.09	1.26	1.51	0.11	0.08
Lysine	93.9	122	10.7	0.09	1.26	1.52	0.13	0.11
Methionine	27.5	34.5	2.99	0.14	1.12	1.28	0.11	0.19
Phenylalanine	42.5	39.3	2.93	0.46	0.89	0.81	0.03	0.11
Threonine	102	145	17.3	0.10	2.64	3.39	0.30	0.17
Valine	87.0	107	7.58	0.09	1.59	1.85	0.11	0.09
NEAA	205	244	21.4	0.23	0.40	0.48	0.04	0.26
Alanine	41.0	45.6	6.17	0.62	1.30	1.43	0.19	0.77
Aspartate	−0.73	−0.25	3.40	0.92	−0.002	0.002	0.04	0.95
Cysteine	0.09	2.50	0.73	0.03	−0.04	0.48	0.14	0.02
Glutamate	69.5	74.4	7.58	0.67	0.36	0.38	0.04	0.83
Glycine	−8.55	−3.75	9.04	0.71	−0.65	−0.30	0.63	0.66
Proline	16.0	24.7	2.87	0.05	0.20	0.31	0.04	0.05
Serine	42.1	50.4	4.81	0.27	0.84	0.96	0.11	0.40
Tyrosine	45.9	50.9	2.69	0.21	1.04	1.13	0.04	0.12
BCAA	286	358	27.4	0.09	1.43	1.75	0.11	0.07
Total AA	871	1,078	80.6	0.10	0.91	1.10	0.07	0.09
Group 1^2)^	143	159	8.20	0.21	1.00	1.07	0.04	0.17
Group 2^2)^	568	725	58.2	0.09	1.61	2.00	0.13	0.07

AA, amino acids; SEM, standard error of the mean; RFI, residual feed intake; EAA, essential AA; NEAA, non-essential AA; BCAA, branched-chain AA.

1) Uptake (g/d) = arteriovenous difference (g/L) × mammary plasma flow (L/d).

2) Group 1, sum of methionine, phenylalanine, tyrosine, and histidine; Group 2, sum of arginine, valine, isoleucine, leucine, lysine, and threonine.

## References

[1] Connor EE, Hutchison JL, Van Tassell CP, Cole JB (2019). Defining the optimal period length and stage of growth or lactation to estimate residual feed intake in dairy cows. J Dairy Sci.

[2] Seymour DJ, Canovas A, Chud TCS (2020). The dynamic behavior of feed efficiency in primiparous dairy cattle. J Dairy Sci.

[3] Koch RM, Swiger LA, Chambers D, Gregory KE (1963). Efficiency of feed use in beef cattle. J Anim Sci.

[4] Liu E, VandeHaar MJ (2020). Relationship of residual feed intake and protein efficiency in lactating cows fed high- or low-protein diets. J Dairy Sci.

[5] Xi YM, Wu F, Zhao DQ (2016). Biological mechanisms related to differences in residual feed intake in dairy cows. Animal.

[6] Macdonald KA, Pryce JE, Spelman RJ (2014). Holstein-Friesian calves selected for divergence in residual feed intake during growth exhibited significant but reduced residual feed intake divergence in their first lactation. J Dairy Sci.

[7] Djouvinov DS, Todorov NA (1994). Influence of dry matter intake and passage rate on microbial protein synthesis in the rumen of sheep and its estimation by cannulation and a non-invasive method. Anim Feed Sci Technol.

[8] National Research Council (2001). Nutrient requirements of dairy cattle.

[9] Xie Y, Wu Z, Wang D, Liu J (2019). Nitrogen partitioning and microbial protein synthesis in lactating dairy cows with different phenotypic residual feed intake. J Anim Sci Biotechnol.

[10] Arriola Apelo SI, Knapp JR, Hanigan MD (2014). Invited review: Current representation and future trends of predicting amino acid utilization in the lactating dairy cow. J Dairy Sci.

[11] Horwitz W, Latimer GW (2005). Official methods of analysis of AOAC International.

[12] Chen XB, Gomes MJ (1992). Estimation of microbial protein supply to sheep and cattle based on urinary excretion of purine derivatives: an overview of technical details.

[13] Rahmatullah M, Boyde TRC (1980). Improvements in the determination of urea using diacetyl monoxime; methods with and without deproteinisation. Clin Chim Acta.

[14] Mackle TR, Dwyer DA, Bauman DE (1999). Effects of branched-chain amino acids and sodium caseinate on milk protein concentration and yield from dairy cows. J Dairy Sci.

[15] Valadares RFD, Broderick GA, Valadares Filho SC, Clayton MK (1999). Effect of replacing alfalfa silage with high moisture corn on ruminal protein synthesis estimated from excretion of total purine derivatives. J Dairy Sci.

[16] Gargallo S, Calsamiglia S, Ferret A (2006). Technical note: a modified three-step *in vitro* procedure to determine intestinal digestion of proteins. J Anim Sci.

[17] Mepham TB (1982). Amino acid utilization by lactating mammary gland. J Dairy Sci.

[18] Rius AG, McGilliard ML, Umberger CA, Hanigan MD (2010). Interactions of energy and predicted metabolizable protein in determining nitrogen efficiency in the lactating dairy cow. J Dairy Sci.

[19] Bequette BJ, Backwell FRC, Crompton LA (1998). Current concepts of amino acid and protein metabolism in the mammary gland of the lactating ruminant. J Dairy Sci.

[20] Doepel L, Lapierre H (2010). Changes in production and mammary metabolism of dairy cows in response to essential and nonessential amino acid infusions. J Dairy Sci.

[21] Mackle TR, Dwyer DA, Ingvartsen KL, Chouinard PY, Ross DA, Bauman DE (2000). Effects of insulin and postruminal supply of protein on use of amino acids by the mammary gland for milk protein synthesis. J Dairy Sci.

[22] Omphalius C, Lapierre H, Guinard-Flament J, Lamberton P, Bahloul L, Lemosquet S (2019). Amino acid efficiencies of utilization vary by different mechanisms in response to energy and protein supplies in dairy cows: Study at mammary-gland and whole-body levels. J Dairy Sci.

[23] Richardson CM, Baes CF, Amer PR (2020). Determining the economic value of daily dry matter intake and associated methane emissions in dairy cattle. Animal.

[24] Martineau R, Ouellet DR, Kebreab E, White RR, Lapierre H (2017). Relationships between postruminal casein infusion and milk production, and concentrations of plasma amino acids and blood urea in dairy cows: a multilevel mixed-effects meta-analysis. J Dairy Sci.

[25] Wohlt JE, Clark JH, Derrig RG, Davis CL (1977). Valine, leucine, and isoleucine metabolism by lactating bovine mammary tissue. J Dairy Sci.

[26] Li F, Yin Y, Tan B, Kong X, Wu G (2011). Leucine nutrition in animals and humans: mTOR signaling and beyond. Amino Acids.

[27] Nichols K, Kim JJM, Carson M, Metcalf JA, Cant JP, Doelman J (2016). Glucose supplementation stimulates peripheral branched-chain amino acid catabolism in lactating dairy cows during essential amino acid infusions. J Dairy Sci.

[28] Lapierre H, Berthiaume R, Raggio G (2005). The route of absorbed nitrogen into milk protein. Anim Sci.

[29] Sun HZ, Zhao K, Zhou M, Chen Y, Guan LL (2019). Landscape of multi-tissue global gene expression reveals the regulatory signatures of feed efficiency in beef cattle. Bioinformatics.

[30] Salleh SM, Mazzoni G, Lovendahl P, Kadarmideen HN (2018). Gene co-expression networks from RNA sequencing of dairy cattle identifies genes and pathways affecting feed efficiency. BMC Bioinformatics.

